# Growth and Duration of Inflammation Determine Short- and Long-Term Outcome in Very-Low-Birth-Weight Infants Requiring Abdominal Surgery

**DOI:** 10.3390/nu15071668

**Published:** 2023-03-29

**Authors:** Corinna Peter, Abdulmonem Abukhris, Julia Brendel, Carolin Böhne, Bettina Bohnhorst, Sabine Pirr

**Affiliations:** 1 Department of Paediatric Pneumology, Allergology and Neonatology, Hannover Medical School, Carl-Neuberg-Str. 1, 30625 Hannover, Germany; 2 Department of Paediatric Surgery, Hannover Medical School, Carl-Neuberg-Str. 1, 30625 Hannover, Germany

**Keywords:** childhood outcomes, cholestasis, enterostomy, intestinal complication, longitudinal growth restriction, neurodevelopmental delay, parenteral nutrition, preterm infants

## Abstract

Necrotizing enterocolitis (NEC), spontaneous intestinal perforation (SIP) and meconium-related ileus (MI) requiring surgical intervention are associated with a high risk of severe short- and long-term complications in very-low-birth-weight (VLBW) infants including poor growth, cholestasis and neurodevelopmental impairment. This retrospective study aimed to identify risk factors for such complications in a cohort of 55 VLBW preterm infants requiring surgery with enterostomy creation due to NEC, SIP or MI. Long-term follow-up was available for 43 (78%) infants. Multiple regression analyses revealed that the duration of inflammation and longitudinal growth determined the risk of cholestasis and neurodevelopmental outcome at 2 years corrected age independent of the aetiology of the intestinal complication. Direct bilirubin increased by 4.9 μmol/L (95%CI 0.26–9.5), 1.4 μmol/L (95%CI 0.6–2.2) and 0.8 μmol/L (95%CI 0.22–1.13) with every day of elevated (Interleukin-6) IL-6, (C-reactive protein) CrP and parenteral nutrition. The mental development index at 2 years corrected age decreased by 3.8 (95%CI −7.3–−0.36), 0.4 (95%CI 0.07–0.80) and 0.3 (95%CI 0.08–0.57) with every day of elevated IL-6 and every 1 point decrease in weight percentile at discharge and 2 years. These data stress the importance of optimal timing for the initial surgery in order to prevent prolonged inflammation and an early reversal of the enterostomy in case of poor growth or insufficient enteral nutrition.

## 1. Introduction

Neonatal abdominal disorders such as necrotizing enterocolitis (NEC), spontaneous intestinal perforation (SIP) and meconium-related ileus (MI) requiring surgical intervention are associated with a high mortality in very-low-birth-weight (VLBW) preterm neonates. Surviving infants face severe short- and long-term morbidities such as poor growth, cholestasis and neurodevelopmental impairment [1]. The latter was reported to affect up to 61% of surviving infants at a corrected age (CA) of 18 to 36 months [2]. The risk of cholestasis increases significantly with prolonged parenteral nutrition and any cause of systemic inflammation [3,4], which are both frequently present in infants suffering from surgical NEC, SIP or MI. In these infants, cholestasis is mostly transient and only rarely accompanied by liver failure [3]. However, it is associated with poor growth [5], which in turn is a predictor for an adverse neurodevelopmental outcome in preterm infants [6]. A better understanding of risk factors associated with short- and long-term morbidities of surgically treated NEC, SIP and MI is needed to guide treatment strategies for improved outcomes and to reliably counsel parents.

This study aimed to identify risk factors that help to assess the probability for adverse short- and long-term outcomes in VLBW infants suffering from surgical NEC, SIP or MI.

## 2. Methods

### 2.1. Study Design and Population

In a single-centre retrospective study, all in- or outborn preterm neonates treated at the neonatal intensive care unit (NICU) of Hannover Medical School between January 2006 and December 2018 were included when fulfilling the following criteria: birth weight < 1500 g (VLBW) and surgery with the creation of an enterostomy due to NEC, SIP or MI. NEC, SIP and MI were diagnosed at the presence of clinical criteria (including bowel distension, abdominal tenderness and/or discoloration, feeding intolerance, regurgitation, bilious or stool vomiting) by ultrasound and radiography as well as laboratory evaluation. The indication for surgery was given in case of fulminant progress and/or signs of intestinal perforation or suspected perforation. Clinical data of those infants were obtained from the hospital information system. Infants with major congenital malformations or syndromes were excluded from the analysis. Furthermore, infants who were either transferred to another hospital for further treatment or died within 14 days after surgery were omitted due to missing data on weight gain and nutrition after initial surgery as well as short-term morbidity.

Data collection included baseline patient characteristics, details on the primary surgery with enterostomy creation, any re-laparotomy if required and the reversal of the enterostomy, as well as enteral and parenteral nutrition prior to and after surgery, number of central-line-associated bloodstream infections (CLABSI) and blood transfusions. Furthermore, laboratory values to assess the occurrence of cholestasis (direct bilirubin) and the inflammatory response (C-reactive protein (CrP) and Interleukin-6 (IL-6)) after primary surgery were evaluated. The normal level for direct bilirubin is <5 μmol/L; therefore, cholestasis was defined as a direct bilirubin > 35 μmol/L [4] and infants were routinely monitored until discharge. CrP and IL-6 serum levels were routinely measured at an interval of 1 to 2 days until negative to guide antibiotic treatment. Only elevated levels in association with the initial surgery or surgeries in case of the necessity of multiple procedures were considered for the analysis. Values > 10 mg/L for CrP and >150 ng/L for IL-6 were considered elevated [7,8]. In order to analyse weight gain after the initial surgery, body weight measurements were collected on a weekly basis. Weekly weight gain was expressed as a percentage of weight change relative to the weight at the time of initial surgery. We did not adjust for any weight gain from oedema directly following surgery as this to some extent was expected in all infants included in our study. Finally, data on growth throughout the onward treatment as well as growth and neurodevelopmental outcomes at 2 years of CA were collected. Growth was assessed using weight, head circumference (HC) and corresponding percentiles according to Voigt et al. and Brandt preterm growth charts up to 42 weeks postmenstrual age and beyond, respectively [9,10]. Any changes in weight or HC percentiles over time were calculated against birth percentiles and expressed as delta percentile (delta percentile = current percentile–birth percentile).

### 2.2. Long-Term Outcome Measures

According to the German National Neonatal Follow-up Program, infants were routinely invited at 2 years CA for the assessment of growth and neurodevelopmental outcome by an experienced neonatologist or paediatric neurologist. All age-related outcomes were corrected for prematurity. For the evaluation of cognitive development, the German version of the Bayley Scales of Infant Development III (Bayley-III) was applied [11]. If Bayley was not feasible due to a lack of cooperation, language barrier or major disabilities, the Griffiths Mental Development Scales edition II was used [12]. Cognitive development was expressed as Bayley-III score or Griffiths development quotient, which are age standardized (MDI, mental development index, normal population mean 100, standard deviation 15). Moderate or severe neurodevelopmental delay was defined as a Bayley-III score or Griffiths development quotient < 85 [13]. Patients who were lost to follow-up were not considered.

### 2.3. Statistical Analysis

Data were evaluated in the pseudonymised form using Excel 2016^®^ (Microsoft Corporation, Redmond, WA, USA) and GraphPad^®^ (GraphPad Prism Version 9.3.1 for Windows, GraphPad Software, San Diego, CA, USA) and tested for Gaussian distribution using the Shapiro–Wilk normality test. Continuous variables were presented as median and range, and categorical variables as frequencies and percentages. For analyses, Mann–Whitney *U* tests, Chi^2^ tests and Kruskal–Wallis tests for multiple comparisons were applied. Spearman’s correlation coefficient was used to estimate the relationship between individual variables. To identify influencing factors on the 2-year outcome or cholestasis, multiple linear regression analyses were performed using the MDI or the maximum direct bilirubin as the dependent variable and building linear models taking clinical characteristics of the infants into account. As potential influencing factors, we included the gestational age, birth weight, maximum inflammation values and duration of inflammation data and number of CLABSIs in both models. In the cholestasis model, we additionally included duration of parenteral nutrition, out-born status and number of transfusions and surgeries, and in the 2-year outcome model intraventricular haemorrhage and delta weight percentile data for initial surgery, discharge and 2-year CA. In order to remove the insignificant variables, we applied a backward elimination process to iteratively remove the least important variables until only significant factors remained in the model. The significance level was set at 0.05.

## 3. Results

### 3.1. Patient Characteristics

**Figure 1:**
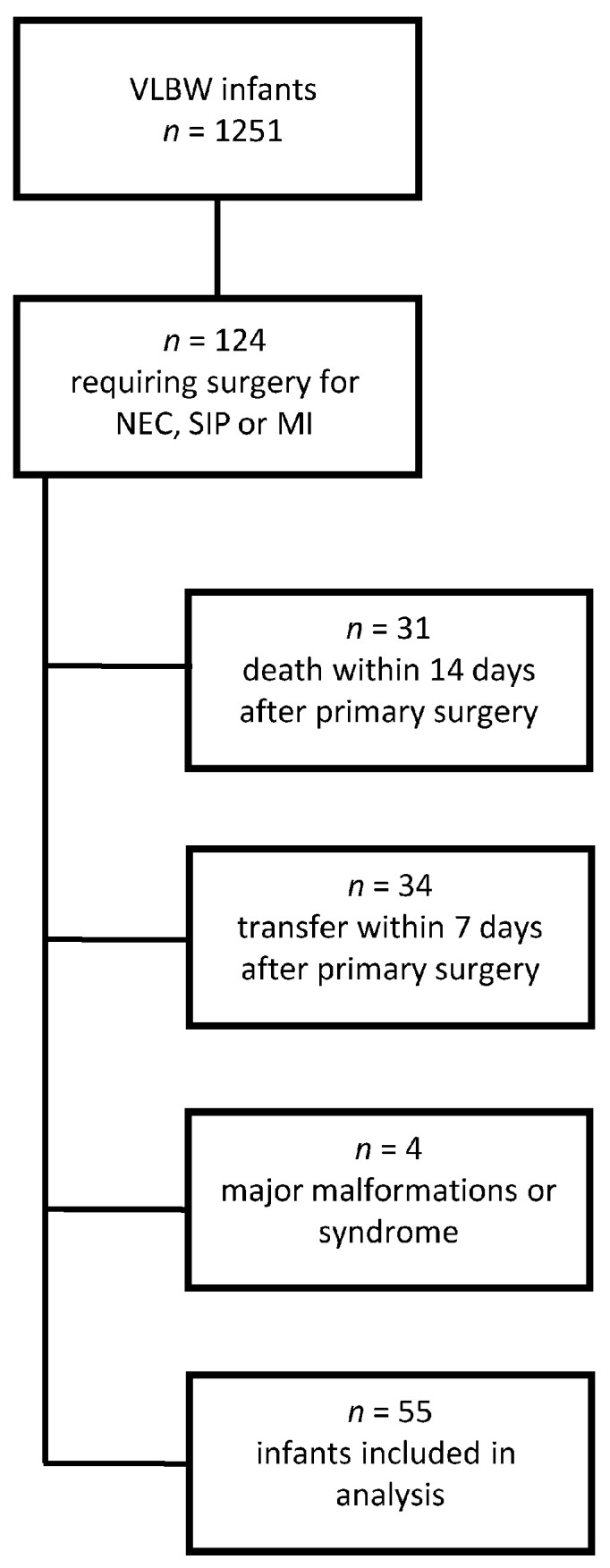
Flow chart of infant recruitment.

Between January 2006 and December 2018, a total of 1251 VLBW preterm infants were treated on the NICU of Hannover Medical School. Of those, 63 patients (5.0%) developed NEC, with 45 infants requiring surgery. SIP was diagnosed in 53 infants (4.2%), and MI in 26 patients (2.1%), all of whom underwent surgery. All operated infants received an enterostomy. Sixty-nine of these surgically treated infants (55.6%) were excluded from further analyses due to an early transfer to another hospital, death or major malformations (one congenital disorder of glycosylation (CDG) syndrome; one Cornelia-de-Lange-syndrome; two complex cardiac defects). Figure 1 provides further details on patient recruitment.

Of the thirty-one infants who died within 14 days after the initial surgery, nineteen died in the course of a fulminant NEC, eleven suffered from SIP and one from MI. Ten of those infants died from septic complications caused by the SIP/MI, one infant suffered from a fungal sepsis and one infant died from respiratory complications (pulmonary emphysema). No further deaths occurred during in-house treatment. Of the fifty-five infants included in the analyses, sixteen were treated for NEC, thirty-two infants for SIP and seven infants received a surgical intervention for MI. None of the infants with MI were diagnosed with cystic fibrosis. Ten infants required the creation of more than one enterostomy in the same or a second surgical session (five infants received a jejuno- and an ileostomy, two an ileo- and a colostomy and three infants two ileostomies each). In the remaining forty-five infants a single enterostomy was created, with three infants received a jejunostomy, forty an ileostomy and two a colostomy. Information regarding the neurodevelopmental outcome at a CA of 2 years was available for 43 out of the 55 remaining infants (78.2%). Twelve infants were lost to follow-up.

NEC was diagnosed at a median postnatal age of 16.5 days (range 3 to 50 days). SIP and MI were diagnosed significantly earlier at a postnatal age of 9.0 days (range 2 to 40 days) (*p* = 0.0071). All out-born infants (*n* = 20) were transferred to the Hannover Medical School for the diagnosis of NEC (*n* = 7), SIP (*n* = 11) or MI (*n* = 3) prior to the initial surgery with a median age of 9 days (range 1–55 days). Baseline characteristics of all patients are presented in Table 1.

No significant differences were found for gestational age, weight and head circumference at birth, rate of cholestasis and neurodevelopmental delay at 2 years CA between infants suffering from NEC, SIP or MI (Table 2).

### 3.2. Cholestasis

Cholestasis occurred in 20 patients (36.4%) and was diagnosed at a median age of 59 days (range 10–156) with a maximum direct bilirubin of 80 μmol/L (median, range 37–180 μmol/L). The characteristics and clinical parameters of infants with and without cholestasis and their statistical analyses are summarized in Table 3.

**Figure 2:**
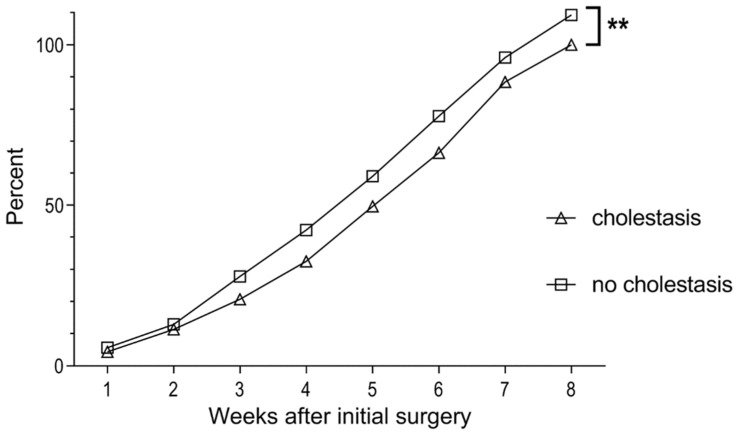
Median weight gain per week after initial surgery in percent of weight at surgery for infants with and without cholestasis. Paired *t*-test, ** *p* = 0.0011.

Of note, outborn infants showed a significantly longer duration of CrP elevation after the initial surgery (median duration and range in days outborn 13 (0–104), inborn 7 (0–38), *p* = 0.025). Infants with cholestasis showed a significantly poorer weight gain starting 3 weeks after the initial surgery (Figure 2).

A multiple regression analysis including all 55 infants revealed that direct bilirubin values were determined by the duration of IL-6 and CrP elevation and of parenteral nutrition after the initial surgery. Every additional day of parenteral nutrition increased direct bilirubin by 0.8 μmol/L (95%CI 0.22–1.13, *p* = 0.004). Furthermore, direct bilirubin rose by 1.4 μmol/L (95%CI 0.6–2.2, *p* = 0.001) and 4.9 μmol/L (95%CI 0.26–9.5, *p* = 0.039) for every day with an elevated CrP and IL-6, respectively.

### 3.3. Outcome at 2 Years CA

In total, the follow-up data of 43 patients (78.2%) were available at a CA of 2 years. The outcome parameters of neurodevelopment and growth for these infants are summarized in Table 4.

Table 5 shows a comparison of the clinical parameters and characteristics of the initial hospital stay and growth parameters up to 2 years CA between infants with and without neurodevelopmental delay. Significant differences between both groups were found for weight development at the initial surgery and at 2 years CA, as well as for the duration but not the extent of IL-6 elevation after initial surgery as a marker for inflammation. A multiple regression analysis including all 43 infants revealed that poor growth during the initial hospital stay and up to 2 years CA as well as a longer duration of IL-6 elevation after the initial surgery significantly determined MDIs at 2 years CA. The MDI decreased by 0.4 (95%CI 0.07–0.80, *p* = 0.021) and 0.3 (95%CI 0.08–0.57, *p* = 0.011) for every 1 point decrease in weight percentile at discharge and 2 years CA, respectively. Furthermore, every day with an elevated IL-6 reduced the MDI at 2 years CA by 3.8 (95%CI −7.3–−0.36, *p* = 0.032).

## 4. Discussion

This study analysed a cohort of VLBW preterm infants requiring surgery with the creation of an enterostomy due to NEC, SIP or MI. The aim of the study was to identify risk factors for short- and long-term complications, in order to improve treatment strategies and counselling of parents. We found that in these particular patients, the duration of the inflammation and longitudinal growth determined the risk of cholestasis and neurodevelopmental outcome at 2 years CA independent of the aetiology of the intestinal complication.

The rate of cholestasis in preterm infants varied greatly with the degree of prematurity, birth weight and the presence of additional risk factors. Previous studies identified those infants at the greatest risk of developing a cholestasis with a birth weight below 750 g, those lacking enteral feeding and receiving prolonged parenteral nutrition and those with intestinal complications including NEC and SIP [4]. The risk of cholestasis continuously increases with the duration of parenteral nutrition [4]. Accordingly, in our cohort, infants with cholestasis presented with a longer duration of parenteral nutrition after the initial surgery. Furthermore, infants without cholestasis showed a better weight gain in the first weeks after the initial surgery. This might be due to the higher number of infants with sufficient enteral nutrition among those patients [14]. Consequently, infants suffering from insufficient enteral nutrition and developing cholestasis might benefit from an early reversal of the enterostomy in order to enable enteral nutrition, avoid prolonged parenteral nutrition and subsequent cholestasis and allow optimal growth [15]. Moreover, it has previously been reported that next to prolonged parenteral nutrition, cholestasis itself is associated with growth delay [5]. The risk of developing cholestasis is further aggravated by the presence of any cause of a systemic inflammatory response [4]. Our study confirmed these findings as infants with cholestasis showed a significantly higher incidence of CLABSIs, a greater need for re-laparotomies, higher maximum CrP values and a longer duration of CrP and IL-6 elevation after the initial surgery. However, regression analyses revealed that the risk of cholestasis was not determined by the number of re-laparotomies or the maximum elevation of CrP but by the duration of the systemic inflammation. Furthermore, infants with cholestasis were more likely to be outborn with the need for transfer for the initial surgical treatment. This might result in a significantly longer duration of inflammation, and therefore a higher rate of cholestasis in these infants. The duration of IL-6 elevation showed the strongest elevating effect on direct bilirubin levels. In this regard, the time between the indication for surgery and surgery itself should be kept as short as possible. In our cohort, gestational age and birth weight did not show a significant association with the risk of cholestasis as reported previously [4]. This is most likely due to the very restricted patient cohort included in our analyses. Furthermore, we did not find an association for the lack of enteral feeds prior to the initial surgery with the later risk of cholestasis. Veenstra et al. reported a decreased risk of later cholestasis if preterm infants received enteral nutrition within the first week of life [16]. However, in our cohort, all but six infants received enteral feeds prior to the initial surgery, two of whom later developed cholestasis. Finally, in our cohort, infants with cholestasis more frequently required a blood transfusion, a risk factor that has previously been reported by other authors [17]. However, it might merely be an indicator of the degree of illness in these infants as the regression analysis did not reveal a significant effect on direct bilirubin values.

Preterm infants have long been recognized as a population at high risk for neurodevelopmental impairment. Especially in the context of declining mortality rates, attention has increasingly turned to the measurement of long-term morbidities and associated risk factors [1]. Several factors have been identified as predictors of an adverse neurodevelopmental outcome including low gestational age and birth weight, sex, bronchopulmonary dysplasia, intraventricular haemorrhage and growth restriction [1,18]. Furthermore, the risk of severe neurodevelopmental delay is significantly increased in infants suffering from NEC, SIP or MI [19,20]. Therefore, 25 to 61% of surviving infants showed severe impairment at 18 to 36 months CA with the highest risks among extremely-low-birth-weight (ELBW) infants requiring surgical treatment. The numbers vary widely as the definitions used for neurodevelopmental delay and for NEC differ between studies [2]. In our cohort, the proportion of infants with moderate or severe neurodevelopmental delay at a CA of 2 years was at the upper range compared to other studies (58%) [2]. This could be explained by our restricted inclusion criteria (VLBW infants with NEC, SIP or MI who required surgical intervention). These criteria probably also masked the previously described association of gestational age and low birth weight with the neurodevelopmental outcome in our cohort [1,18]. Furthermore, we did not find significant correlations between severe intraventricular haemorrhage or bronchopulmonary dysplasia and neurodevelopmental delay most likely due to the low number of included patients. However, our analyses revealed a significant association between adverse neurodevelopmental outcome and extrauterine growth restriction not only during onward treatment but up to a CA of 2 years. Accordingly, De Rose et al. recently reported that longitudinal extrauterine growth restriction is a predictor for adverse neurodevelopmental outcome in preterm infants born before 30 weeks of gestation [6]. However, longitudinal growth does not differ between infants with NEC or SIP or compared to infants without these complications at a CA of 2 or 6 years [19,21].

Finally, in our study, the duration of IL-6 elevation as a biomarker for systemic inflammation was significantly associated with the neurodevelopmental outcome at a CA of 2 years. Several clinical and animal studies identified inflammation as an important contributor to adverse neurodevelopment [22,23,24]. The mechanisms of systemic inflammation-induced neuronal injury have not been fully elucidated, but systemic inflammation is known to disrupt the blood–brain barrier and incite a local inflammatory response in the brain [23,24]. Several previous studies have assessed the relationship between inflammation-related proteins in neonatal blood and subsequent neurodevelopmental outcomes [25,26,27]. They reported several significantly elevated cytokines and chemokines (i.e., IL-6) in cord blood and blood samples of the first weeks of life in preterm infants that later developed neurodevelopmental impairment [26,27]. An integrative review of 37 studies identified an elevation of IL-6, IL-1β, IL-8 and TNF-α during the first 3 weeks of life as potential, independent predictors of brain injury and neurodevelopmental impairment [28]. In our study, only measures of IL-6 and CrP were available for analyses as these were part of the routine work-up at expected inflammation/infection. Lee et al. found an association between CrP and plasma TNF-α levels during systemic inflammatory episodes induced by clinical infection or NEC with poor neurodevelopmental outcomes among preterm infants born before 30 weeks of gestation [25]. IL-6 was not included in their analyses. The ELGAN Study included repeated protein analyses during the first 4 weeks of life at weekly intervals and showed a stronger association when inflammation markers were elevated on more than one time point [26,27]. However, it remained unclear whether elevated inflammation markers on two or more time points reflected repeated episodes or a sustained elevation. To our knowledge, this is the first study demonstrating that it is not the extent but the duration of IL-6 elevation that determines the risk of neurodevelopmental impairment independent from the aetiology of the intestinal complication in a patient group at exceptionally high risk.

Not surprisingly, none of the infants with MI were diagnosed with cystic fibrosis, as the MI of the preterm infant must be distinguished from the meconium ileus of the term-born infant which often is associated with cystic fibrosis.

The limitations of this study are the retrospective approach restricting available data and the small number of included patients. This is due to the overall low incidence of NEC, SIP and MI in preterm infants and our single-centre approach. However, a multi-centre design carries the bias of treatment variabilities between centres and would have required a treatment alignment before the study. Furthermore, a relatively high number of infants had to be excluded from analysis due to early death or transfer after initial surgery. A further limitation is the use of different test tools for the evaluation of neurodevelopmental delay at 2 years CA, and the relatively short follow-up period, which is too short to gain true insight into the long-term prognosis as specific deficits in neurodevelopment might not emerge until later [19,20].

## 5. Conclusions

This study provides evidence of the high risk of cholestasis and neurodevelopmental delay in VLBW infants requiring surgery with the creation of an enterostomy for NEC, SIP or MI. As risk factors for cholestasis, we identified prolonged parenteral nutrition and sustained inflammation. The latter was also significantly associated with neurodevelopmental delay at a CA of 2 years next to longitudinal growth failure. For the first time, we demonstrated that it is not the extent but the duration of the elevation of the inflammation-associated proteins CrP and IL-6 that determines the risk of cholestasis and neurodevelopmental delay in this patient group regardless of the underlying disease, i.e., NEC, SIP or MI. These data stress the importance of optimal timing for the initial surgery once the indication is given in order to prevent prolonged inflammation as well as for an early reversal of the enterostomy in case of poor growth or insufficient enteral nutrition. Furthermore, they provide guidance for parent counselling at the time of initial diagnosis.

## Figures and Tables

**Table 1:** Baseline characteristics of infants included in the analysis (*n* = 55).

Characteristic	Median (Range) or *n* (%)
Gestational age, weeks	25.3 (23.1–32.3)
Birth weight, grams	710 (315–1450)
Birth weight percentile	30 (1–92)
HC at birth, cm	23 (19–29)
HC percentile at birth	30 (1–80)
Sex, male	36 (65.5)
Out-born	21 (33.2)
Age at primary surgery, days	12 (2–50)
Age at reversal of enterostomy, days	75 (37–280)
Bronchopulmonary dysplasia, moderate or severe	8 (14.5)
Intraventricular haemorrhage > 2°	7 (12.7)

HC, head circumference.

**Table 2:** Clinical characteristics and data availability of the infants included in the analyses stratified for aetiology of the intestinal complication, shown as median and range or *n* and %.

	NEC *n* = 16	SIP *n* = 32	MI *n* = 7	*p* Value ^a^
Gestational age, weeks	25.8 (24.3–32.3)	25.0 (23.1–30.4)	26.0 (23.3–32.0)	0.26
Birth weight, grams	663 (380–1370)	713 (315–1450)	790 (645–1375)	0.69
Head circumference, cm	23.5 (19–29)	22.9 (20–28)	22 (22–28.5)	0.75
Cholestasis	9 (56.3)	10 (31.3)	1 (14.3)	0.11
Data on neurodevelopmental outcome available	12 (75.0)	26 (81.3)	5 (71.4)	0.80
Neurodevelopmental delay at 2 years CA	8 (66.7)	15 (57.7)	2 (40.0)	0.60

^a^ Kruskal–Wallis test. CA, corrected age.

**Table 3:** Characteristics of infants (*n* = 55) with vs. without cholestasis, shown as median and range or *n* and %. Significant differences are marked in bold.

	Cholestasis *n* = 20	No Cholestasis *n* = 35	*p* Value
Gestational age, weeks	25.4 (23.1–31.7)	25.7 (23.3–32.3)	0.71 ^a^
Birth weight, grams	760 (315–1370)	695 (575–1450)	0.77 ^a^
Birth weight percentile	25 (1–92)	30 (5–90)	0.63 ^a^
Sex, male	11 (55.0)	25 (71.4)	0.22 ^b^
Out-born	12 (60.0)	9 (25.7)	**0.01** ^b^
Aetiology of intestinal complication	NEC 9 (45.0) SIP 10 (50.0) MI 1 (5.0)	NEC 7 (20.0) SIP 22 (62.9) MI 6 (17.1)	0.47 ^b^
CrP max, mg/L	145.5 (21–275)	75 (1.7–401)	**0.03** ^a^
Duration of CrP elevation, days	19 (2–104)	6 (0–20)	**<0.0001** ^a^
IL-6 max, ng/L	2040 (18–15,900)	153.5 (30–11,000)	0.07 ^a^
Duration of IL-6 elevation, days	4 (0–10)	0 (0–7)	**0.0005** ^a^
Enteral nutrition prior to initial surgery	18 (90.0)	31 (88.6)	0.87 ^b^
Duration of parenteral nutrition after initial surgery, days	24 (3–96)	10 (4–59)	**0.001** ^a^
Type of enteral nutrition after initial surgery, exclusive formula	11 (55.0)	22 (62.9)	0.57 ^b^
Duration between creation and reversal of enterostomy, days	63.5 (33–251)	58 (18–121)	0.50 ^a^
Short bowel syndrome prior to reversal of enterostomy ^c^	4 (20.0)	2 (5.7)	0.10 ^b^
Number of CLABSIs	1 (0–4)	0 (0–2)	**0.04** ^a^
Number of blood transfusions	9 (5–36)	5 (1–16)	**0.0005** ^a^
Number of laparotomies before reversal of enterostomy	2 (1–9)	1 (1–7)	**0.03** ^a^
Age at initial surgery, days	15 (7–40)	10 (2–50)	0.09 ^a^

^a^ Mann–Whitney *U* test. ^b^ Chi^2^ test. ^c^ Malabsorptive condition caused by the lack of functional small intestine due to a very proximal enterostomy and the subsequent continuous dependency on parenteral nutrition NEC, necrotizing enterocolitis; SIP, spontaneous intestinal perforation; MI, meconium-related ileus; CrP, C-reactive protein; IL-6, interleukin-6; CLABSIs, central-line-associated bloodstream infections.

**Table 4:** Outcome at 2 years CA (*n* = 43).

Outcome Parameter	Median (Range) or *n* (%)
Neurodevelopmental delay, moderate or severe	25 (58.1)
MDI	90 (50–120)
Body weight, grams	10,800 (8300–15,700)
Weight percentile	20 (1–83)
Delta weight percentile ^a^	−6 (−70–+47)
HC, cm	46.8 (38.6–52)
HC percentile	4 (1–80)
Delta HC percentile ^a^	−19 (−69–+50)

^a^ Delta percentiles were calculated against birth measurements. MDI, mental development index; HC, head circumference.

**Table 5:** Clinical parameters and characteristics of infants (*n* = 43) with vs. without neurodevelopmental delay at 2 years CA, shown as median and range or *n* and %. Significant differences are marked in bold.

	Neurodevelopmental Delay *n* = 25	No Neurodevelopmental Delay *n* = 18	*p* Value
Gestational age, weeks	25.0 (23.1–32.3)	25.6 (23.3–32.0)	0.82 ^b^
Aetiology of intestinal complication	NEC 8 (32.0)	NEC 4 (22.2)	0.60 ^c^
	SIP 15 (60.0)	SIP 11 (61.1)	
	MI 2 (8.0)	MI 3 (16.7)	
Outborn	7 (28.0)	5 (27.8)	0.99 ^c^
Sex (male)	19 (76.0)	11 (61.1)	0.29 ^c^
Age at initial surgery, days	9 (2–50)	12 (2–37)	0.87 ^b^
Number of transfusions	7 (1–36)	6.5 (1–32)	0.93 ^b^
Number of CLABSIs	0 (0–3)	0.5 (0–4)	0.45 ^b^
Number of laparotomies before reversal of enterostomy	2 (1–9)	1 (1–5)	0.24 ^b^
Bronchopulmonary dysplasia, moderate or severe	5 (20.0)	1 (5.5)	0.18 ^c^
Intraventricular haemorrhage > 2°	5 (20.0)	2 (11.1)	0.44 ^c^
Maximum CrP, mg/L	114 (7–474)	121 (1.7–327)	0.96 ^b^
Duration of CrP elevation, days	9.5 (0–104)	9.5 (0–38)	0.62 ^b^
Maximum IL-6, ng/L	688.5 (18–15,900)	1889 (34–11,000)	0.99 ^b^
Duration of IL-6 elevation, days	2 (0–10)	0 (0–5)	**0.048** ^b^
*Growth percentiles at birth*			
Weight, grams	690 (315–1450)	788 (510–1375)	0.38 ^b^
Weight percentile	30 (1–90)	40 (9–90)	0.15 ^b^
HC, cm	22.5 (19–28)	23.3 (21–28.5)	0.53 ^b^
HC percentile	30 (1–80)	40 (15–70)	0.40 ^b^
*Growth percentiles at initial surgery*			
Weight percentile	15 (1–50)	20 (1–50)	0.70 ^b^
Delta weight percentile ^a^	−15 (−66–0)	−9 (−45–5)	**0.02** ^b^
HC percentile	8 (1–40)	9 (1–40)	0.70 ^b^
Delta HC percentile ^a^	−23.5 (−69–0)	−15 (−55–0)	0.18 ^b^
*Growth percentiles at discharge*			
Weight percentile	4 (1–85)	3.5 (1–30)	0.96 ^b^
Delta weight percentile ^a^	−24 (−70–27)	−23.5 (−80–−8)	0.15 ^b^
HC percentile	1 (1–98)	1 (1–50)	0.74 ^b^
Delta HC percentile ^a^	−20 (−70–28)	−23 (−69–10)	0.60 ^b^
*Growth percentiles at 2 years CA*			
Weight percentile	20 (1–60)	30 (1–83)	0.57 ^b^
Delta weight percentile ^a^	−18.5 (−70–45)	6.5 (−69–47)	**0.03** ^b^
HC percentile	2 (1–80)	4 (1–26)	0.89 ^b^
Delta HC percentile ^a^	−16 (−69–50)	−27.5 (−66–0)	0.25 ^b^

^a^ Delta percentiles were calculated against birth measurements. ^b^ Mann–Whitney *U* test. ^c^ Chi^2^ test. NEC, necrotizing enterocolitis; SIP, spontaneous intestinal perforation; MI, meconium-related ileus; CLABSIs, central-line-associated bloodstream infections; CrP, C-reactive protein; IL-6, interleukin-6; HC, head circumference; CA, corrected age.

## Data Availability

The data presented in this study are available on reasonable request from the corresponding author. The data are not publicly available due to restrictions in the data access agreement by the institution’s Ethical Board and Data Protection Board.

## Abbreviations

CA: corrected age; CLABSI: central-line-associated bloodstream infection; CrP: C-reactive protein; HC: head circumference; IL-6: Interleukin-6; MDI: mental development index; MI: meconium-related ileus; NEC: necrotizing enterocolitis; NICU: neonatal intensive care unit; SIP: spontaneous intestinal perforation; VLBW: very-low-birth-weight.
